# Pure Chromic Acid

**Published:** 1887-07

**Authors:** 


### Pure Chromic Acid.

A new form of chromic acid, prepared by Merck, and alleged to be absolutely free from sulphuric acid, and hence very slowly deliquescent in dry air, is now procurable. The acid is in the form of small dry red crystals, readily soluble in water and in alcohol. It is said that the acid in this form does not spread over the surrounding tissue, and is therefore better suited for use in places where it would be dangerous to employ the ordinary acid.

THE students of the University of Paris numbered nearly 11,000 last year. Of these, 3,786 were studying law, and 3,696 were studying medicine, while only 35 were studying theology. The female students numbered 167.

A poison antidote table says that equal parts of calcined magnesia, powdered charcoal, and hydrated peroxide of iron, in a sufficient quantity of water, is a general antidote for poisoning, for use when the poison is not known. It is a perfectly harmless and simple preparation.

THE TEETH FROM A MEDICO-LEGAL ASPECT.—The dientification of dead bodies and criminals is sometimes a matter of much perplexity. For instance: the features of a dead body may be distorted or destroyed; the clothes changed or unrecognizable; and no ordinary circumstances left to make identification clear. Some such case occured in Michigan. A man was found in a lake murdered. As the coroner was about dismissing the case as "unidentified," the neighboring dentist had the curiosity to look into the mouth. In a moment he said: "I have a chart of that mouth in my office," and though he could not then remember the name, he soon found it by referring to his chart-book. It resulted in tracing the murderer.—*Med. and Surg. Rep.*

CIRCULATION NOT ESSENTIAL TO LIFE.—Circulation of vital fluid is not absolutely essential to life. A tooth, an egg, a seed can live without circulation. If an artificial tooth is placed in the jaw, the gum and alveolar process shrink away from it; it will not adhere to the new object, from want of sympathy. But the natural tooth adheres to the surrounding parts, though it is not inclined to ulcerate, suppurate, or experience any other mode of being rejected as a foreign body. An egg has life, and maintains it without circulation. And a seed placed outside the influence of heat, moisture, and atmospheric action, will germinate after a hundred years. A tooth appears to have no membranes and vessels penetrating it, like other forms of bone, nor is it subject, as above stated, to the changes incidental to other living parts; it has no such pores or membranes; yet it will often adhere to and form connection with its cavity, if timely replaced after removal. —*Medical World.*

